# Revisiting *Ehrlichia ruminantium* Replication Cycle Using Proteomics: The Host and the Bacterium Perspectives

**DOI:** 10.3390/microorganisms9061144

**Published:** 2021-05-26

**Authors:** Isabel Marcelino, Philippe Holzmuller, Ana Coelho, Gabriel Mazzucchelli, Bernard Fernandez, Nathalie Vachiéry

**Affiliations:** 1 CIRAD, UMR ASTRE, F-97170 Petit-Bourg, Guadeloupe, France; nathalie.vachiery@cirad.fr; 2 ASTRE, CIRAD, INRAE, Université de Montpellier (I-MUSE), 34000 Montpellier, France; philippe.holzmuller@cirad.fr; 3 Unité TReD-Path, Institut Pasteur de la Guadeloupe, 97183 Les Abymes, Guadeloupe, France; 4 CIRAD, UMR ASTRE, 34398 Montpellier, France; bernard.fernandez@cirad.fr; 5 Instituto de Tecnologia Química e Biológica, Universidade Nova de Lisboa, Av. da República, 2780-157 Oeiras, Portugal; varela@itqb.unl.pt; 6 GIGA-Proteomics, B4000 Liege, Belgium; Gabriel.mazzucchelli@uliege.be

**Keywords:** *Ehrlichia ruminantium*, endothelial cells, host response, immunomodulation, bacterial life cycle, differential protein expression, infection biomarkers, virulence factors

## Abstract

The Rickettsiales *Ehrlichia ruminantium*, the causal agent of the fatal tick-borne disease Heartwater, induces severe damage to the vascular endothelium in ruminants. Nevertheless, *E. ruminantium-*induced pathobiology remains largely unknown. Our work paves the way for understanding this phenomenon by using quantitative proteomic analyses (2D-DIGE-MS/MS, 1DE-nanoLC-MS/MS and biotin-nanoUPLC-MS/MS) of host bovine aorta endothelial cells (BAE) during the in vitro bacterium intracellular replication cycle. We detect 265 bacterial proteins (including virulence factors), at all time-points of the *E. ruminantium* replication cycle, highlighting a dynamic bacterium–host interaction. We show that *E. ruminantium* infection modulates the expression of 433 host proteins: 98 being over-expressed, 161 under-expressed, 140 detected only in infected BAE cells and 34 exclusively detected in non-infected cells. Cystoscape integrated data analysis shows that these proteins lead to major changes in host cell immune responses, host cell metabolism and vesicle trafficking, with a clear involvement of inflammation-related proteins in this process. Our findings led to the first model of *E. ruminantium* infection in host cells in vitro, and we highlight potential biomarkers of *E. ruminantium* infection in endothelial cells (such as ROCK1, TMEM16K, Albumin and PTPN1), which may be important to further combat Heartwater, namely by developing non-antibiotic-based strategies.

## 1. Introduction

*Ehrlichia ruminantium* is a Gram-negative, obligate intracellular bacterium that belongs to the Rickettsiales order. It is transmitted by *Amblyomma* ticks and causes Heartwater, an acute and fatal disease of ruminants, present throughout sub-Saharan Africa and some islands in the Indian Ocean and the Caribbean (from where it threatens to invade the Americas) [1]. Clinical signs and lesions are associated with functional injury to the vascular endothelium, causing increased vascular permeability and leading to hydropericardium, hydrothorax, oedema and congestion of the brain and lungs in ruminants [2].

In vivo, *E. ruminantium* is the only *Ehrlichia* sp. that mainly infects vascular endothelial cells, covering small- and medium-sized blood vessels in infected animals. In vitro, *E. ruminantium* can be propagated experimentally by serial passages in culture in different cell types, but most reliably in finite culture of endothelial cells (as reviewed in [1]). In endothelial cells, *E. ruminantium* has a developmental cycle characterized by two distinct forms, the elementary body (EB) and the reticulate body (RB) [3]. EBs are small (0.2–0.5 μm in diameter), being the free extracellular infectious forms of the bacterium. After cell invasion, they reside within intracytoplasmic inclusions (called morulae), where they convert into the larger (0.75–2.5 μm) intracellular non-infectious, metabolically active, reticulate bodies (RBs). RBs multiply by binary fission, rapidly filling the inclusion, which expands in size. RBs re-condense back into EBs towards the end of the cycle, being released after host cell lysis [3]. The relation between the stage of development and time post-infection depends on the *E. ruminantium* strain and its adaptation to in vitro culture conditions [4,5,6].

*E. ruminantium* has a small genome [7] and, to survive and replicate within host cells, it has developed complex mechanisms to hijack host cellular functions [6,8,9]. Nevertheless, many important and basic cell biology questions regarding *E. ruminantium*–host cell interaction remain unaddressed. Unraveling these fine molecular mechanisms is key to understand the pathological dysfunctions of Heartwater disease and then, to define improved therapeutics strategies.

Differential proteomics has proven to be a powerful tool to identify molecular pathways involved in specific cell functions [10,11,12]. Preliminary work performed by our group revealed that major changes in BAE biochemical pathways are observed at the bacteria’s mid-exponential phase of development (72 h post-infection) [13]. Herein, we applied differential quantitative proteomics to characterize the variations in host cell protein abundance in response to infection to *E. ruminantium* in vitro, during the bacterium life cycle. For this, three proteomic approaches were used to monitor the expression level of both the host cell and the bacterium proteins, at different times post-infection. First, differential in-gel electrophoresis (2D-DIGE)-MS/MS was used to map and monitor proteins differentially expressed between infected and non-infected cells from early to mid-exponential *E. ruminantium* replication phase (24–72 hpi). Then, 1DE-nLC-MS/MS allowed us to monitor proteins’ modulation throughout the bacterium life cycle (from 24 to 120 hpi), and a specific biotin crosslinking enrichment protocol was used to focus on surface/membrane proteins at *E. ruminantium* mid-exponential replication phase (72 hpi). All acquired data were integrated into a biological network using Cytoscape, to gain new insights into host cell physiology and metabolism during *E. ruminantium* infection. We then draw a schematic putative model for *E. ruminantium* penetration and development within bovine host endothelial cells, including the host and bacterial factors involved in the infection process in vitro. The defined interactions provide valuable information to identify new ways to interfere with the infectious process and to further improve Heartwater control.

## 2. Materials and Methods

### 2.1. Experimental Design and Statistical Rationale

Five independent biological replicates per time of infection (TOI) were produced either for non-infected bovine aorta endothelial (BAE) cells (used as control) or BAE infected with the virulent *E. ruminantium,* strain Gardel (isolated in Guadeloupe, Taxon identifier 302409, NCBI: txid302409). To detect and identify proteins differentially expressed between non-infected and *E. ruminantium*-infected BAE, 2D-DIGE-MS/MS, 1DE-nLC-MS/MS and a biotin crosslinking enrichment protocol were used (Supplementary Figure S1). The 2D-DIGE-MS/MS approach was used to map and quantitatively assess the proteins and the proteoforms differentially expressed between infected and non-infected cells at 24, 48 and 72 hpi. The 1DE-nLC-MS/MS strategy was used to identify the proteins differentially expressed in BAE cells throughout the complete *E. ruminantium* life cycle (from 24–120 hpi), and the biotin protocol was used to evaluate modifications in host cell surface/membrane proteins at only 72 hpi. For 2D-DIGE-MS/MS experiments, the five independent biological replicates per TOI were used, image analyses were performed using Decyder software and spot-normalized volume was used to select statistically significant differentiated spots between *E. ruminantium*-infected and non-infected BAE cells analyzed in the experiment (fold-change, ANOVA, false discovery rate and power value). For 1DE-nLC-MS/MS experiments, three independent replicates per TOI (from 24 to 120 hpi) previously used for 2D-DIGE-MS/MS analyses were used. Results from 1DE-nLC-MS/MS experiments were analyzed using MaxQuant software package (v 1.5.0.0) [14], followed by Perseus (v1.5.3.0). For the biotin crosslinking enrichment protocol, three independent replicates (for both infected and non-infected BAE) were harvested at 72 hpi. All these details are summarized in Supplementary Figure S1 and described below in more detail. The mass spectrometry proteomics data have been deposited to the ProteomeXchange Consortium via the PRIDE [15] partner repository with the dataset identifier PXD026092.

### 2.2. Samples’ Preparation

#### 2.2.1. Cell Culture and *E. ruminantium* Infection

Bovine aorta endothelial (BAE) cells were isolated from a Creole cow. Briefly, BAE were isolated from the aorta by treatment with collagenase, then cultured to constitute a primary bank and then working banks in pyramidal subcultures. BAE and *E. ruminantium* Gardel virulent strain (isolated in Guadeloupe, Taxon identifier 302409, NCBI: txid302409) were routinely propagated as described elsewhere [4]. Briefly, BAE were maintained at 37 °C and 5% of CO_2_ in BHK-21 medium (Glasgow modified 1, Gibco, USA), supplemented with 10% FBS (Gibco, USA) and 2 mM glutamine. *E. ruminantium* samples up to passage 13 were used throughout this study, as they are known to be highly infectious [5]. For the experiments, several biological replicates were produced in parallel in 75 cm^2^ T-flasks: 5 for infected and 5 for non-infected cells (used as control), with sample harvesting at every 24 h post-infection (from 24 to 120 hpi). After cells reached confluence (100% of the area of the flask covered by the cell monolayer), BAE were infected with approximately 400 *E. ruminantium*/cell to ensure that the cells were synchronously infected [4]. A complete medium refeed was made at 24 and 72 hpi, for both infected or non-infected cells [4]. Infection progress was followed using an inverted light microscope (Leica DM IL LED Fluo, Leica, Germany): at 24 hpi, the cells appeared to be highly infected (foci of swollen and refractive cells in the cell monolayer), with few morula observables at 72 hpi, no cell lysis at 96 hpi (but with many morulae inside the host cells) and up to 80% of host cell lysis at 120 hpi, with the release of EBs. All infection kinetics were similar (data not shown). Non-infected cells, used as negative controls for each time point of infection, kept their regular cobblestone pattern throughout the kinetics and sub-culturing routines.

#### 2.2.2. Sample Harvesting at Different Time Points Post-Infection

For sample harvesting, at each time point post-infection from 24 to 96 hpi, and for corresponding non-infected cells, cell monolayer was washed with ice-cold Tris-sucrose buffer (33 mM Trizma, 250 mM sucrose, pH 7.4) containing anti-proteases (cOmplete™ Protease Inhibitor Cocktail, Roche, Germany) and anti-phosphatases (PhosSTOP™, Roche, Germany) and harvested using a cell scraper. Cell suspension was centrifuged at low speed (120× *g*, 15 min, 4 °C) and the pellet was directly frozen at −80 °C. At 120 hpi, when cell lysis occurs, cell debris and EBs were harvested directly without a washing step using a cell scraper, anti-proteases and anti-phosphatases (stated above) were added to the suspension and this was centrifuged at 20,000× *g*, 30 min, 4 °C, and the pellet was immediately frozen at −80 °C.

#### 2.2.3. Protein Extracts Preparation and Quantification

To prepare protein extracts, cell pellets were snap-frozen in liquid nitrogen, and DIGE buffer (7 M urea, 2 M Thiourea, 4% CHAPS, 30 mM Tris, pH 8.5, 250 µL per sample) was added. Samples were then sonicated on ice for an efficient protein extraction and DNA shearing. Samples were centrifuged at 20,000× *g*, 20 min at 4 °C, and contaminants (salts, DNA, RNA, etc.) were removed using the 2D Clean-up Kit (GE Healthcare, Sweden). Protein quantification was performed using the 2D Quant kit (GE Healthcare, Sweden).

### 2.3. Proteomics Analyses

#### 2.3.1. 2D-DIGE-MS/MS

##### Isoelectric Focusing (IEF)

Protein separation of five biological replicates per TOI (24–72 hpi) was performed. To compare protein differentially expressed between infected and non-infected cells but also compare proteins differentially expressed across time, a total of 15 gels were made, each of them including an internal standard stained with Cy2 dye composed of a pool of all biological replicates at 24, 48 and 72 h. For each TOI, each protein sample of *E. ruminantium—*infected and uninfected BAE (50 μg)—was labeled with 400 pmol of Cy3 or Cy5, and Cy2 was used as an internal calibrator. After an incubation step on ice for 30 min in the dark, the labeling reaction was stopped with 10 mM lysine. For each gel, Cy5- and Cy3-labeled proteins plus the Cy2-labeled internal standard were mixed with 130 μL rehydration buffer (7 M urea, 2 M thiourea, 2% (*w*/*v*) CHAPS, 130 mM DTT, 2% IPG buffer pH 3–10, all from GE Healthcare, Sweden). The labeled protein mixture was applied to Immobiline DryStrip strips (24 cm, pH 3–11 NL; GE Healthcare, Sweden) overnight for strip rehydration. IEF was then performed using the IPGphor system (GE Healthcare, Sweden), using cup loading and manifold. The protocol consisted of a sequence of 5 steps as follows: a step-and-hold running condition at 150 V (3 h), followed by a second step-and-hold at 300 V (3 h), then a gradient step at 1000 V (6 h), another at 10,000 V for 1 h and a final step-and-hold at 10,000 V (3 h), with a total of 40.75 Vh (16 h). All were performed at 75 µA/strip at 20 °C.

##### SDS-PAGE

After IEF, the IPG strips were equilibrated: the samples were first reduced in equilibration buffer (50 mM Tris pH 8.8, 6 M urea, 30% (*v*/*v*) glycerol and 2% (*w*/*v*) SDS) supplemented with DTT (10 mg/mL), followed by alkylation in equilibration buffer supplemented with iodocetamide (25 mg/mL). Each equilibration step took 15 min, under slow agitation, at room temperature. The IPG strips were then embedded in a precast gel (GE Healthcare, Sweden) and sealed into place using 0.5% (*w*/*v*) agarose sealing solution. The SDS-PAGE was performed using an Ettan DALT six system (GE Healthcare, Sweden) with a discontinuous system of SDS electrophoresis buffer (25 mM Tris-HCl, pH 8.3, 192 mM glycine, 0.1% (*w*/*v*) SDS) in the bottom chamber and SDS electrophoresis buffer (50 mM Tris-HC pH 8.3, 384 mM glycine, 0.2% (*w*/*v*) SDS) in the top chamber overnight at 150 V, 12 mA/gel, 2 W/gel.

##### Image Analysis and Protein Spot Picking

After the electrophoretic run, gels were scanned at different wavelengths depending on the dye used (Cy2, Cy3 and Cy5) with the high-resolution scanner Typhoon FLA 9500 (GE Healthcare, Sweden). Gel images were then analyzed using the various modules of Decyder 2-D 7.2 software (GE Healthcare, Sweden). The difference in-gel analysis (DIA) module was used to determine the optimal and average spot detection settings. Gel-to-gel spot matching was performed using the BVA module. All 15 gel images were then processed using the biological variation analysis (BVA) module, and Cy2-labeled pooled standard was used to normalize spot intensity within each gel. The spot map with the greatest number of detected spots (3140 spots) was set as the master gel, and the module was then used for automated spot matching across all the gels. Gel matching quality was manually verified, and landmarks were added wherever improved matching quality was needed. For identification of differential proteins, each sample spot map was assembled into the appropriate experimental group: condition 1 corresponds to cell status (infected or non-infected cells) and condition 2 to time of infection (24, 48, 72 hpi), and the average ratio fold-change was calculated. A base set was established using only spots that were matched on greater than 80% of the spot maps. Considering the ratio infected vs. non-infected at each TOI, only spots having fold-change ≥1.2 or ≤−1.2 and *p*-value < 0.05 were considered differentially expressed, with each spot being manually verified for an acceptable three-dimensional characteristic protein profile and for adequate material for subsequent mass spectrometry identification. About 199 protein spots were found to be differentially expressed. These spots were automatically excised by the Ettan spot picker (GE Healthcare) and transferred to a 96-well collecting plate containing 120 µL of a 10% ethanol solution. Plates with excised spots were stored at −20 °C until further use.

##### In-Gel Digestion for 2DE-Spots and Protein Identification by MALDI-TOF/TOF

The proteins of interest were digested into their component peptides with trypsin and eluted from the gel plugs, as described previously [5]. Briefly, spots were de-stained, reduced with DTT, alkylated with iodoacetamide and dried in a speedvac. Gel pieces were rehydrated with digestion buffer (50 mM NH4HCO3) containing trypsin (6.7 ng/µL; Promega, Madison, WI, USA) and incubated overnight at 37 °C. The buffered peptides were acidified with formic acid, desalted and concentrated using homemade reversed phase microcolumns (POROS R2, Applied Biosystems, Foster City, CA, USA). The peptides were eluted onto a MALDI plate using a matrix solution that contained 5 mg/mL α-cyano-4-hydroxycinnamic acid dissolved in 50% (*v*/*v*) ACN/0.1% (*v*/*v*) formic acid.

Protein identification was performed on a 4800 MALDI-TOF/TOF instrument (Applied Biosystems, Framingham, MA, USA) with 4000 series explorer v3.5 software in both MS and MS/MS mode. Each MS spectrum was obtained in a result-independent acquisition mode with a total of 800 laser shots per spectrum, with internal calibration using Angiotensin II (1046.2 Da), Angiotensin I (1296.5 Da), Neurotensin (1672.9 Da), ACTH [1,2,3,4,5,6,7,8,9,10,11,12,13,14,15,16,17] (2093.5 Da) and ACTH [18,19,20,21,22,23,24,25,26,27,28,29,30,31,32,33,34,35,36,37,38,39] (2465.7 Da; PepMix1, Laserbio labs, France). Fifteen signal-to-noise (s/n) best precursors from each MS spectrum were selected for MS/MS analysis, starting with the most intense peak and ending with the least intense peak. MS/MS analyses were performed using CID (collision-induced dissociation) assisted with air, using a collision energy of 1 kV and a gas pressure of 1 × 10^−6^ Torr. A total of 1400 laser shots were collected for each MS/MS spectrum with the peak detection minimum S/N setting at 20.

Protein identification was performed with the software Protein-Pilot, using *Bos taurus* (48,272 entries) and *E. ruminantium* (4149 entries) Uniprot databases (release 2019_04), and peptide modification carbamidomethylation was set as fixed and oxidation (M) as variable.

#### 2.3.2. Label-Free Quantitative 1DE-nLC-MS/MS

Proteins (10 µg) from 3 biological replicates of paired non-infected and *E. ruminantium*-infected BAE cells at 5 time-points post-infection (24, 48, 72, 96 and 120 hpi; 30 samples in total) were separated on SDS-PAGE gels (12% polyacrylamide, Mini-PROTEAN^®^ TGX™ Precast Gels, Bio-Rad, CA, USA) and stained with Protein Staining Solution (Euromedex, Souffelweyersheim, France). Gel lanes were cut into 3 gel pieces and de-stained with 50 mM triethylammonium bicarbonate (TEABC) and three washes in 100% acetonitrile. After protein reduction (with 10 mM dithiothreitol in 50 mM TEABC at 56 °C for 45 min) and alkylation (55 mM iodoacetamide TEABC at room temperature for 30 min), proteins were digested in-gel using trypsin (0.9 µg/band, Gold, Promega, Madison, MA, USA), as previously described [16]. Digest products were dehydrated in a vacuum centrifuge and reduced to 2 μL. The generated peptides were analyzed online by nano-flowHPLC–nanoelectrospray ionization using an Orbitrap Elite mass spectrometer (Thermo Fisher Scientific, Waltham, MA, USA) coupled to an Ultimate 3000 HPLC (Thermo Fisher Scientific). Desalting and pre-concentration of samples were performed online on a Pepmap^®^ pre-column (0.3 × 10 mm, Dionex, France). A gradient consisting of 0–40% B for 60 min and 80% B for 15 min (A = 0.1% formic acid, 2% acetonitrile in water; B = 0.1% formic acid in acetonitrile) at 300 nL/min was used to elute peptides from the capillary reverse-phase column (0.075 × 150 mm, Acclaim Pepmap 100^®^ C18, Thermo Fisher Scientific). Eluted peptides were electro-sprayed online at a voltage of 2 kV into an Orbitrap Elite mass spectrometer. A cycle of one full-scan mass spectrum (400–2000 *m*/*z*) at a resolution of 120,000 (at 400 *m*/*z*), followed by 20 data-dependent MS/MS spectra, was repeated continuously throughout the nanoLC separation. All MS/MS spectra were recorded using normalized collision energy (33%, activation Q 0.25 and activation time 10 ms) with an isolation window of 2 *m*/*z*. Data were acquired using the Xcalibur software (v 2.2). For all full scan measurements with the Orbitrap detector, a lock-mass ion from ambient air (445.120024 *m*/*z*) was used as an internal calibrant as described [17].

Analysis of MS data was performed using the MaxQuant software package (available online: https://maxquant.net/). Tandem mass spectra (MS/MS) were searched by the Andromeda search engine [18] against the UniProtKB Proteome UP000000533 database for *E. ruminantium* (strain Gardel) (release 2016_02 identical to release 2019_04, 948 entries) and the UniProtKB Reference Proteome UP000009136 database for BAE (release 2016_02 identical to release 2019_04, 24,112 entries) using the following parameters: enzyme specificity was set as Trypsin/P, and a maximum of two missed cleavages and a mass tolerance of 0.5 Da for fragment ion were applied. A second database of known contaminants provided with the MaxQuant suite was also employed. The “match between runs” option was checked. Oxidation (M) was specified as variable modification and carbamidomethyl (C) as fixed modification. Database searches were performed with a mass tolerance of 20 ppm for precursor ion for mass calibration, and with a 4.5 ppm tolerance after calibration. The maximum false peptide and protein discovery rate was specified as 0.01. Seven amino acids were required as minimum peptide length.

The MaxQuant software generates several output files that contain information about identified peptides and proteins. Representative protein ID in each protein group was automatically selected using the in-house developed Leading tool (v2.1, [19]).

Quantification was performed using standard parameters: we only considered proteins with at least 2 peptides identified/quantified for further analysis, after elimination of reverse and contaminant entries. The Perseus software (v 1.5.3.0) enabled protein quantification (label-free quantification) via the iBAQ intensities (the intensities of the precursor peptides that map to each protein are summed together and divided by the number of theoretically observable peptides, which is considered to be all tryptic peptides between 6 and 30 amino acids in length. This operation converts a measure that is expected to be proportional to mass (intensity) into one that is proportional to molar amount (iBAQ) [20]) and performed subsequent statistical analysis for pairwise comparisons of samples from *E. ruminantium*-infected vs. non-infected BAE, by creating a matrix analytical workflow for parallel analysis of mass spectrometry data for the proteins identified in the bovine host cell or bacterium, respectively [21]. Working on MS signal intensity-based analysis, proteins were filtered out if they were not quantified in at least all replicates from one condition. For BAE proteins’ analysis, a scatter plot based on a Student’s T-test (two tailed T-test, unequal variances) of triplicate MS analyses was performed on log^2^-transformed values using the Perseus toolbox. Criteria used to classify the proteins were the Student’s T-test difference calculated by Perseus (difference between the two compared conditions of the mean log^2^-transformed value for triplicate MS/MS analyses), and the Student’s T-test *p*-value. Results were filtered using the following combination of criteria: −1 ≥ |Welch *t*-test difference| > 1 and -Log *p*-value > 2. For the *E. ruminantium* proteins’ analysis, a profile plot analysis of triplicate MS analyses was performed on log^2^-transformed values using the Perseus toolbox. Criteria used to classify the proteins were the reference profiles of significant protein relative amount during the infection kinetics.

#### 2.3.3. Biotin Enrichment Protocol

Experiments were performed with 3 biological replicates for *E. ruminantium*-infected and non-infected BAE cells at 72 hpi (6 samples in total). Surface protein biotinylation was performed according to Turtoi and co-workers [22]. Briefly, after culture medium removal, cells were washed 3 times in cold PBS (4 °C). Freshly prepared biotin (10 mg per 50 mL PBS, pH 8) was then added to cell monolayers and left at room temperature for 5 min on a rocking platform. To stop labeling reaction, Tris-HCl (1 M) was added to a final concentration of 50 mM. After buffer removal, cell monolayers were washed once with cold PBS containing oxidized L-Glutathione (GSSG, Sigma). Cell monolayers were then scraped from the T-flask using culture medium containing EDTA and GSSG. Cells were centrifuged and washed once with culture medium containing EDTA and GSSG. The resulting pellet was stored at −80 °C until further processing. Cell pellet was lysed in 2.5 mL lysis buffer (1 mL Nonidet P40 (NP40), 1 mL EDTA 5 M, 2% SDS, 0.5 mM GSSG and 37 mL of PBS, pH 7.0), sonicated (10 s), incubated on ice for 30 min and centrifuged at 16,000 g for 10 min (4 °C). The supernatant was further collected. All lysates from the three solubilization steps were finally pooled together. Protein immunoblotting using the secondary antibody neutravidin-HRP (Pierce) was used to confirm sample labeling [22] (data not shown).

Afterwards, 600 µL of protein extract from each replicate was mixed with 200 μL Streptavidin (SA) resin (Pierce, Rockford, IL, USA) for 120 min at RT on a rocking platform. The biotinylated proteins were eluted 2 times with 0.2 mL of 100 mM dithiothreitol (DTT) and incubated at 60 °C for 30 min, alkylated with 1 M 2-chloroacetamide for 30 min in the absence of light. One internal standard consisting of biotin oval burin (ovalbumin) was added to each replicate. Proteins were then precipitated in the presence of 20% trichloroacetic acid (TCA) overnight (4 °C) and washed two times with ice-cold acetone. The amount of protein from each of the 6 replicates was assessed using the EZQ Protein Quantification Kit (Invitrogen). The isolated biotinylated proteins were then solubilized in 30 µL of 50 mM NH4HCO3 and digested (1:50 protease/protein ratio) overnight using trypsin (Promega, Madison, WI, USA) (37 °C). The digestion was further extended for 4 h by addition of fresh trypsin (using a dilution 1:100).

Fifteen microliters (0.175 µg/µL; 3.5 µg) of peptides originating from biotinylated protein samples were desalted using C18 ZipTip pipette tips (Millipore, Billerica, MA, USA). Afterwards, the peptide mixtures were desiccated and dissolved in 10 μL of 100 mM ammonium formate buffer (pH 10). To the dissolved samples, 4.2 µL of 3 internal standard mix was added to the different samples: MassPREP Digestion Standard Mixture 1 was included in the 3 non-infected biological replicates and the MassPREP Digestion Standard Mixture 2 (Waters, USA) to the 3 *E. ruminantium*-infected replicates, containing equimolar mix of yeast alcohol dehydrogenase, rabbit glycogen phosphorylase b, bovine serum albumin and yeast enolase, and a final concentration in 15 μL sample was adjusted to 135 fmol of yeast alcohol dehydrogenase. From the prepared sample, 9 μL were injected, corresponding to an estimated protein load of 2.5 μg. For the MS analysis, the 2D-nano Aquity UPLC (Waters, USA) was coupled online with the SYNAPT G1 qTOF system (Waters, USA).

The configuration of the 2D-nano UPLC system was the following: first dimension separation column X-Bridge BEH C18 5 μm (300 μm × 50 mm), trap column Symmetry C18 5 μm (180 μm × 20 mm) and analytical column BEH C18 1.7 μm (75 μm × 150 mm) (all Waters). The sample was loaded at 2 μL/min (20 mM ammonium formiate, pH 10) on the first column and subsequently eluted in 5 steps (10%, 14%, 16%, 20% and 65% acetonitrile). Each eluted fraction was desalted on the trap column and subsequently separated on the second analytical column: flow rate 300 nL/min, solvent A (0.1% formic acid in water) and solvent B (0.1% formic acid in acetonitrile), gradient 0 min, 97% A; 90 min, 60% A. The MS acquisition parameters were: data-independent, alternate scanning (MSE) mode, 50–1500 *m*/*z* range, ESI+, V optics, scan time 1 s, cone 30 V and lock mass [Glu1]-Fibrinopeptide B ([M + 2H]2 + 785.8426 *m*/*z*).

Raw data were processed (deconvoluted, deisotoped, protein identification, absolute and relative quantification) using ProteinLynx Global SERVER (PLGS) v2.4 (Waters, USA). The processing parameters were: MS TOF resolution, and the chromatographic peak width was set to automatic, low-/elevated-energy detection threshold to 250/100 counts, identification intensity threshold to 1500 counts and lock mass window to 785.8426 ± 0.30 Da.

To assess the inter-replicate repeatability of the enrichment protocol and further analyses, an internal biotinylated standard was used. This consisted of examining the flow-through of the streptavidin purification step for remaining biotinylated proteins. Overall, the capture of biotinylated proteins was efficient and allowed a good specificity and minor sample loss, as supported by the data obtained. It is noteworthy that in the BIOT fraction, quantitative recoveries of biotinylated ovalbumin were detected. Regarding the reproducibility of the protein digestion and subsequent purification steps, good recoveries of biotinylated ovalbumin were observed in all replicates, indicating that the quantitative and qualitative variability introduced by these steps are within the acceptable limits.

Processed MS spectra were then searched against the *Bos taurus* UniProt database (release 2019_04, 48,272 entries). Peptide modification carbamidomethylation was set as fixed and oxidation (M) as variable. PLGS software calculated the score and the relative ratio of protein expression (infected vs. non-infected). The *p*-value output format (PLGS) ranged from 0 to 1, indicating: (i) 0–0.05, significant downregulation, and (ii) 0.95–1.0, significant upregulation of the respective protein, and both instances indicate a level of certainty ≥95%.

### 2.4. Bioinformatics

To perform the network biological analyses, we used the Cytoscape v3.8.0 software platform [23] combined with different plugins: ClueGO [24], CluePedia [25], BiNGO [26], StringApp [27] and Enrichment Map [28]. The ClueGO (v2.5.7) plugin was used for enrichment analysis of DEPs based on Gene Ontology (GO) term and Kyoto encyclopedia of genes and genomes (KEGG) pathway data. CluePedia (v 1.5.7) was used to explore the functional network. Terms with *p* < 0.05 and a κ score of 0.4 were regarded as significantly enriched terms. For functional analysis of proteins significantly downregulated or upregulated by *E. ruminantium* (q < 0.05), enrichment of GO Biological Process, Molecular Function and Cellular Component terms against a background of all quantitated proteins was determined using BiNGO (v 3.0.4). The threshold for a differentially expressed protein was defined as *p* <0.05. The differential co-expression network was constructed with the absolute value of correlation coefficient ≥ 0.9. Correlation analysis between proteins was measured using Pearson’s correlation coefficient. To account for redundancy between annotations, enriched GO terms were visualized using the Enrichment Map (v3.3.1) with default settings (q-value cut-off of 0.1) and sparse-intermediate connectivity. Clusters were manually labeled to highlight the prevalent biological functions amongst each set of related annotations. To reveal the protein–protein interaction network (PPI) of BAE DEPs, we used the StringApp (v 1.6.0), with default parameters. Using the Kyoto Encyclopedia of Genes and Genomes (KEGG, available online: http://www.kegg.jp) metabolic pathway analysis, the DEPs involved in major Reactome Pathways Functional Interaction (FI) Network were identified. The Reactome Knowledgebase (available online: https://reactome.org) delivers molecular details of transport, metabolism, signal transduction, DNA replication and other cellular processes as an ordered network of molecular transformations—a Reactome is an extended version of a classic metabolic map [29].

## 3. Results

To identify the host proteins modulated by *E. ruminantium* infection, we compared, at different time points, the proteomes of uninfected and *E. ruminantium*-infected BAE cells, using quantitative proteomic tools (2D-DIGE-MS/MS, 1DE-nLC-MS/MS and biotin-nUPLC-MS/MS) (Supplementary Figure S1).

### 3.1. Protein Mapping and Level of Expression during the Early Stages of E. ruminantium Infection

**Figure 1:**
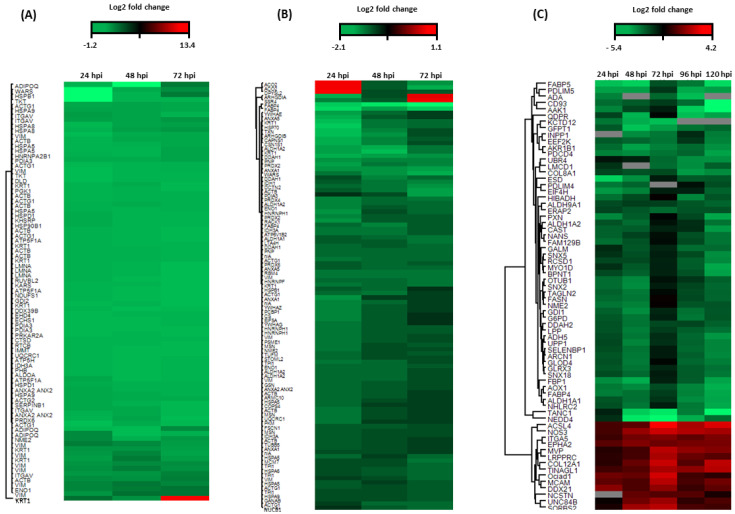
Hierarchical cluster analysis of differentially expressed BAE proteins as detected using different proteomic methodologies at different time points post-infection in *E. ruminantium*-infected BAE cells. 2D-DIGE-MALDI-TOF/TOF: (**A**) for over-expressed proteins and (**B**) for under-expressed proteins. (**C**) 1D-nLC-MS/MS. Fold-change is calculated based on the ratio infected/uninfected BAE cells.

2D-DIGE was used to quantitatively assess proteins and proteoforms differentially expressed between *E. ruminantium*-infected and non-infected BAE from 24 to 72 hpi. Supplementary Figure S2A is a representative example of 2D-DIGE gel images obtained for the five biological replicates (e.g., at 24 hpi). It shows that proteins exhibit isoelectric points (pI) ranging from 3 to 11 and a molecular mass range from 250 to 10 kDa, the most abundant having a medium to high molecular mass. The set of selected differentially expressed proteins (over- or under-expressed by at least 1.2-fold) for each of the experimental group (*E. ruminantium*-infected vs. uninfected BAE cells, at different time-points post-infection) comparisons was used for Principal Component Analysis (PCA) (Supplementary Figure S2B). Supplementary Figure S2B shows that the 30 spot maps clearly clustered into two groups, corresponding to infected (circles in yellow, pink and light blue) or non-infected (circles in black, violet and dark blue). Image analysis with Decyder software revealed that 184 protein spots were found to be significantly differentially expressed from 24 to 72 hpi (*p* < 0.05) between *E. ruminantium*-infected and non-infected cells (Table 1 and Supplementary Table S1). Of all spots analyzed, all were identified as BAE proteins (Supplementary Table S1), with none being of bacterial origin. From those, 84 were found to be over-expressed while 100 are under-expressed (Figure 1A,B, respectively). The Top 5 over-expressed proteins from 24 to 72 hpi include Keratin (KRT1, spot 2054, FC_average_ = 5.4, with a maximum Fc of 13.4 at 72 hpi, Figure 1A, in red), Vimentin (VIM, spot 1025, FC_average_ = 2.8), Alpha-enolase (ENO1, spot 948, FC_average_ = 2.7), Vimentin (VIM, spot 953, FC_average_ = 2.6) and Actin (ACTB, spot 858, FC_average_ = 2.4). The Top 5 under-expressed proteins include Fatty acid-binding protein (FABP4, spots 2956 and 2942, FC_average_ = −2.3 and −2.1 respectively, in red in Figure 1B), N(G), N(G)-dimethylarginine dimethylaminohydrolase (DDAH1, spot 1841, FC_average_ = −1.8), KRT1 (spot 884, FC_average_ = −1.8) and Aldehyde dehydrogenase (ALDH1A2, spot 1256, FC_average_ = −1.8). The proteoforms of specific proteins such as KRT1 (identified in 10 spots) and VIM (identified in 12 spots) were found to be either over- or under-expressed during the infection process (Supplementary Table S1).

### 3.2. E. ruminantium and BAE Differentially Expressed Proteins during the Complete Bacterium Infectious Cycle

The label-free quantitative (LFQ) proteomic analysis permitted us to identify a total of 1876 proteins in BAE (for all the biological replicates, at all time-points). Using Perseus software (v. 1.5.3.0), an infected vs. non-infected paired analysis allowed to evidence the modulation of 131 host proteins (*p* < 0.05) during the complete *E. ruminantium* life cycle (24–120 hpi). Among those, some were found to be over-expressed (*n* = 14) or under-expressed (*n* = 54) (Figure 1C, Supplementary Table S2), while others were only detected in infected (*n* = 36) or non-infected cells (*n* = 27). Among those, we noticed that 12 proteins were detected from 24 to 120 hpi, 14 at 48–120 hpi onwards, 3 at 24–96 hpi and 3 at 24–72 hpi (Supplementary Table S2).

The Top 5 over-expressed BAE proteins during infection are Nitric oxide synthase (NOS3, Fc_average_ = 4.2), Cell surface glycoprotein MCAM (Fc_average_ = 3.5), Integrin alpha-5 (ITGA5, Fc_average_ = 3.2), Tubulointerstitial nephritis antigen-like 1 (TINAGL1, Fc _average_ = 3.2) and Leucine-rich PPR motif-containing protein (LRPPRC, Fc_average_ 3.1) (Supplementary Table S2). The Top 5 downregulated proteins are Aldehyde oxidase 1 (AOX1, Fc_average_ = 5.4), N(G),N(G)-dimethylarginine dimethylaminohydrolase 2 (DDAH2, Fc_average_ = 5.3), 4-trimethylaminobutyraldehyde dehydrogenase (ALDH9A1, Fc_average_ = 4.9), Programmed cell death protein 4 (PDCD4, Fc_average_ = 4.4) and Eukaryotic elongation factor 2 kinase (EEF2K, Fc_average_ = 4.2).

**Figure 2:**
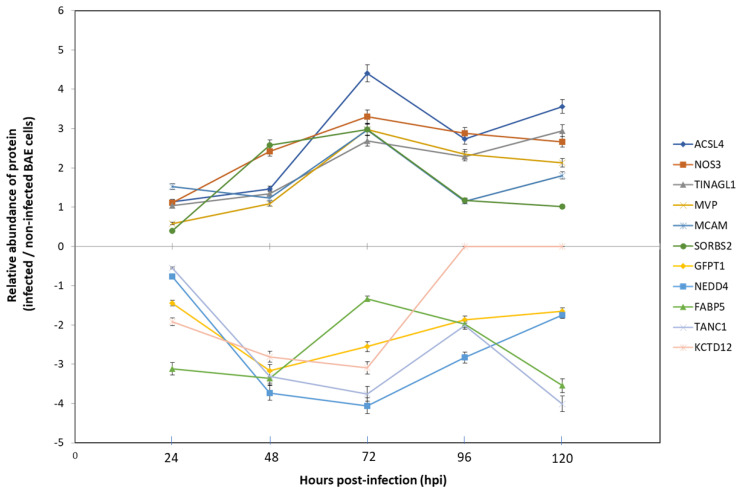
Relative abundance of BAE proteins at different time points after *E. ruminantium* infection. Protein abundance in different conditions was quantified using 1DE-nLC-MS/MS and Perseus (using the ratio infected/non-infected BAE).

Amongst the DEPs, we noticed that the relative abundance of 6 over-expressed proteins, including Acyl-CoA synthetase long-chain family member 4 (ACSL4), Tubulointerstitial nephritis antigen-like 1 (TINAGL1), Nitric oxide synthase (NOS3), Major vault protein (MVP), Cell surface glycoprotein (MCAM) and Sorbin and SH3 domain containing 2 (SORBS2), can substantially vary during infection. For instance, the protein SORBS2 is always over-expressed during the infection, compared to non-infected cells, but a peak in protein abundance up to 7.5-fold is observed at 72 hpi (Figure 1C and Figure 2) when the bacterium is actively replicating. The under-expressed proteins Glutamine-fructose-6-phosphate transaminase 1 (GFPT1), NEDD4 E3 ubiquitin protein ligase (NEDD4), Fatty Acid Binding Protein 5 (FABP5), Tetratricopeptide repeat, ankyrin repeat and coiled-coil containing 1 (TANC1) and Potassium channel tetramerization domain containing 12 (KCTD12) can also vary. TANC1 relative abundance shows a substantial decrease (down to 6.8-fold) at 72 hpi (Figure 1C and Figure 2).

### 3.3. Differentially Expressed Proteins (DEPs) during the Peak of Intracellular Bacterial Replication (72 hpi) 

The biotin enrichment protocol was performed at 72 hpi, which corresponds to the active mid-exponential growth phase of *E. ruminantium* [4]. From this, we identified a total of 2463 protein isoforms (for all replicates of each studied group, *ER*-infected vs. non-infected BAE cells). An infected vs. non-infected paired analysis allowed us to show the modulation of 118 host plasma membrane proteins (*p* < 0.05). Among those, some BAE proteins were found to be under-expressed (*n* = 7), detected only in infected cells (*n* = 104), while others were not detected in infected cells (*n* = 7) (Table 1 and Supplementary Table S3).

At 72 hpi, the following BAE proteins were under-expressed: AP2-associated protein kinase 1 (AAK1), Alpha 2 HS glycoprotein (AHSG), Serum albumin (ALB), Rho GDP-dissociation inhibitor 2 (ARHGDIB), Armadillo repeat containing 10 (ARMC10), Calpain small subunit 1 (CAPNS1), Histone H4 (H4C1), MHC class I antigen (HLA-B), Potassium channel tetramerization domain containing 12 (KCTD12), Urokinase-type plasminogen activator (PLAU), Peptidyl prolyl cis trans isomerase (Ppia) and Ras-related protein Rab-4B. The 104 proteins detected only in *E. ruminantium*-infected BAE cells at 72 hpi are listed in Supplementary Table S3, including: Prion protein (PRNP), Beta lactoglobulin (LGB), Vesicle-associated membrane protein 5 (VAMP5), Fc fragment of IgG receptor and transporter (FCGRT), Voltage-dependent calcium channel alpha-2/delta subunit 1 (cacna2D1), Beta casein (CSN2), Angiotensin converting enzyme Fragment (ACE), Intercellular adhesion molecule-1 (ICAM1), Kappa casein (csn3), Signal transducer and activator of transcription 3 (stat3), Rho GDP dissociation inhibitor 2 (ARHGDIB) and 6-phosphogluconolactonase (PGLS).

The list of proteins that were only detected in non-infected cells includes: Alpha 1B glycoprotein (A1BG), Vitamin D binding protein (GC), Kinesin family member 12 (KIF12), L1CAM protein, Oxidized low-density lipoprotein receptor 1 (OLR1), Alpha 1 acid glycoprotein (ORM1) and Urokinase plasminogen activator receptor (uPAR) (Supplementary Table S3).

### 3.4. DEPs from Host Cells during Infection—An Integrated Analysis

To perform an integrated analysis of the DEPs using Cytoscape, we first created a list of proteins identified using each proteomic methodology (Supplementary Table S4). As identical proteins were identified using different methodologies, we did not use fold-change value but only the status of the proteins (over-expressed, under-expressed, only in infected cells, only in uninfected cells).

#### 3.4.1. Enriched GO Terms

**Figure 3:**
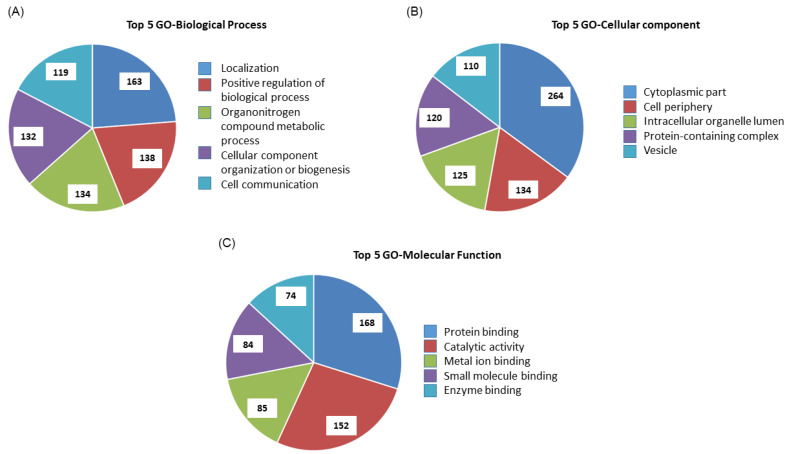
Significantly enriched (Top 5) GO-Biological Process (**A**), GO-Cellular Component (**B**) and GO-Molecular Function (**C**) of DEPs in BAE cells infected by *E. ruminantium*. Number indicates the number of proteins per category.

As the “first topological clustering” method, we used the Cytoscape StringApp plugin [27]. Significantly enriched GO-Biological Process, GO-Cellular Component and GO-Molecular Function terms are presented in Figure 3 and Supplementary Table S5 for DEPs in BAE host cells. The Top 5 biological processes affected by *E. ruminantium* infection (Figure 3A) include: localization (*n* = 163 proteins), positive regulation of biological process (*n* = 138), organonitrogen compound metabolic process (*n* = 134), cellular component organization or biogenesis (*n* = 132) and cell communication (*n* = 119). In the Top 5 GO component group (Figure 3B), DEPs were found to be mainly located in the cytoplasmic part (*n* = 264), cell periphery (*n* = 134), intracellular organelle lumen (*n* = 125), protein-containing complex (*n* = 120) and in vesicles (*n* = 110). Proteins in the Top 5 GO function group (Figure 3C) are capable of catalytic activity (*n* = 152) and binding activities with protein (*n* = 168), metal-ion (*n* = 85), small molecule (*n* = 84) and enzyme (*n* = 74).

#### 3.4.2. Protein–Protein Interaction Network (PPI) and Functional Enrichment Analysis

**Figure 4:**
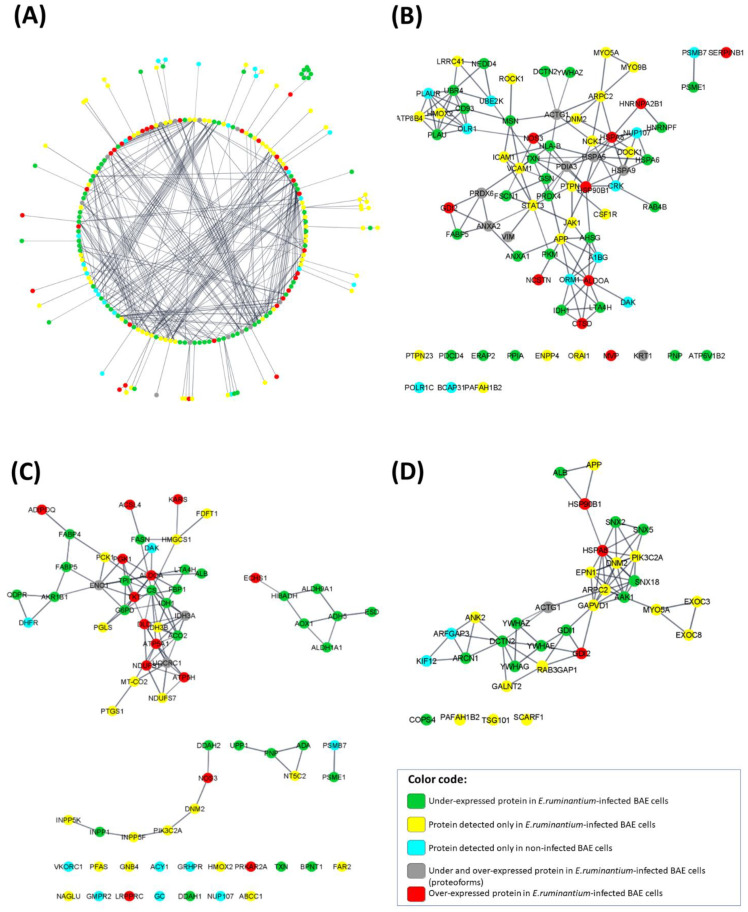
Protein–protein interaction (PPI) network and modular analysis of DEPs identified by 2D-DIGE-MS/MS, 1DE-nanoLC-MS/MS and biotin enrichment protocol (using STRING plugin in Cytoscape with high string confidence = 0.75). (**A**) PPI network was constructed for all the DEPs with a PPI score > 0.8. (**B**–**D**) Modules were obtained from the PPI network based on their Reactome pathways analysis (STRING enrichment plugin in Cytoscape) for immune system (**B**), metabolism (**C**) and vesicle-mediated transport (**D**). Protein names are described in Supplementary Table S4.

The constructed PPI network of DEPs is shown in Figure 4A; from 324 up-loaded proteins in the bioinformatic server (Supplementary Table S4), it was possible to achieve an integrated network with 209 protein nodes and 527 interactions with PPI scores ≥ 0.75, based on the STRING database (2019-04). Thirty-one proteins are over-expressed proteins in infected BAE cells, while seventy proteins are under-expressed proteins. Seventy-five proteins are detected in *E. ruminantium*-infected BAE cells, while twenty-one were detected in non-infected cells. We also included proteins (*n* = 12) whose proteoforms can be either over- or under-expressed in *E. ruminantium*-infected BAE cells.

The Top 5 most enriched Reactome pathways terms were immune System (*n* = 77, FDR = 7.55 × 10^−11^), metabolism (*n* = 73, FDR = 1.43 × 10^−8^), metabolism of proteins (*n* = 57, FDR = 5.50 × 10^-4^), neutrophil degranulation (*n* = 35, FDR = 1.09 ×10^−10^) and vesicle-mediated transport (*n* = 33, FDR = 2.29 × 10^−6^). Due to the redundancy of many proteins between the Reactomes immune system vs. neutrophil degranulation and metabolism vs. metabolism of proteins, we present, in Figure 4B–D, the DEPs present in the 3 Reactome pathways: immune system, metabolism and vesicle-mediated transport, respectively. The “immune system” Reactome pathway contains 77 nodes and 134 interactions, with PPI scores ≥ 0.75 (Figure 4B, Supplementary Table S4). The “metabolism” Reactome pathway contains 73 nodes and 98 interactions, with PPI scores ≥ 0.75 (Figure 4C, Supplementary Table S4). The “vesicle-mediated transport” Reactome pathway contains 33 protein nodes and 68 interactions, with PPI scores ≥ 0.75 (Figure 4D, Supplementary Table S4).

### 3.5. DEPs from E. ruminantium during the Intracellular Infection Cycle

**Figure 5:**
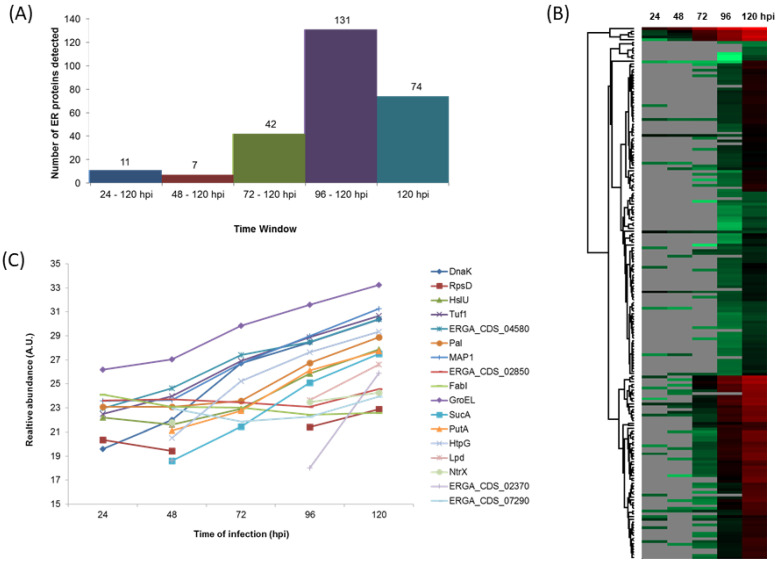
Proteins differentially expressed by *E. ruminantium* in BAE cells from 24 to 120 hpi based on 1DE-nLC-MS/MS. (**A**) Number of *E. ruminantium* proteins detected at each time point post-infection. (**B**) Hierarchical cluster analysis of differentially regulated *E. ruminantium* proteins at each time point post-infection, and (**C**) relative abundance of *E. ruminantium* proteins detected from 24 to 120 hpi.

The use of 1DE-nLC-MS/MS also allowed us to detect 265 *E. ruminantium* proteins, along the bacteria life cycle (Supplementary Table S6), which corresponds to 28% of the *E. ruminantium* Gardel CDS [30]. We detected 11 DEPs from 24 to 120 hpi, 7 from 48 to 120 hpi, 42 from 72 to 120 hpi, 131 from 96 to 120 hpi and 74 at 120 hpi (Figure 5A,B). These differences in protein detection could be related to high levels of BAE proteins, and therefore, dilution of the signal from the peptides of *E. ruminantium* proteins, especially at early time points of the infection process.

Figure 5C shows the relative increase of the 17 *E. ruminantium* proteins detected from 24/48 to 120 hpi: 2-oxoglutarate dehydrogenase E1 component (SucA), 30S ribosomal protein S4 (RpsD), 60 kDa chaperonin (GroEL), ATP-dependent protease ATPase subunit HslU, bifunctional protein PutA, chaperone protein DnaK, chaperone protein HtpG, Dihydrolipoyl dehydrogenase (Lpd), Elongation factor Tu (Tuf1), Enoyl-[acyl-carrier-protein] reductase [NADH] (FabI), Major antigenic protein 1 (MAP1), Nitrogen assimilation regulatory protein NtrX, Peptidoglycan-associated lipoprotein (Pal) and 4 uncharacterized proteins (ERGA_CDS_02370, ERGA_CDS_02850, ERGA_CDS_04580 and ERGA_CDS_07290). Virulence-associated proteins were only detected at 96 (VirB8, VirB2-8, VirB11, VirB4, Ats-1, AnkC) and 120 hpi (AnkA, AnkB, ApxR, VirB2 and VirD4) (Supplementary Table S6).

**Figure 6:**
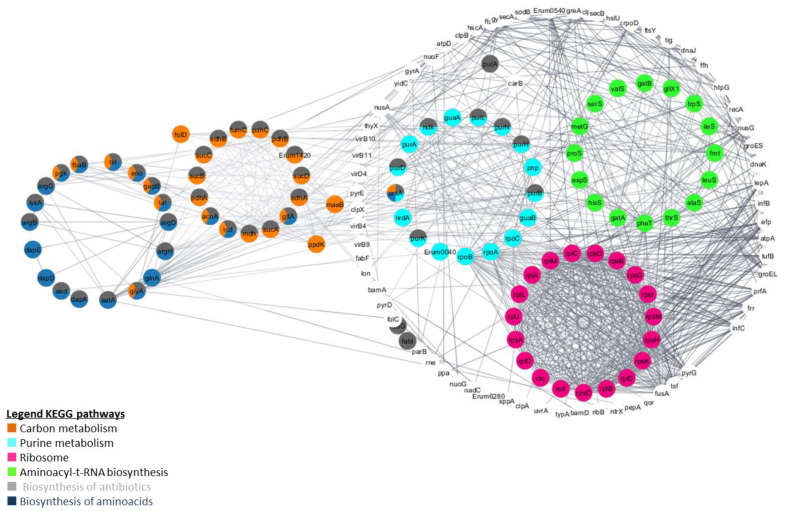
Protein–protein interaction (PPI) network and modular analysis of *E. ruminantium* DEPs identified by 1DE-nLC-MS/MS (using STRING plugin in Cytoscape, high string confidence 0.75)). Modules were obtained from the PPI network based on their KEGG pathways analysis (STRING enrichment plugin in Cytoscape).

To evaluate the PPI interaction network between *E. ruminantium* proteins, we also used the Cytoscape StringApp plugin. The constructed PPI network of DEPs is shown in Figure 6 and contains 169 nodes and 888 interactions, with PPI scores > 0.75, based on the STRING database. To functionally characterize the PPI obtained in Figure 6, we used the StringApp to perform functional enrichment analysis with an FDR threshold of 5%. We then used the filter functionality to remove redundant terms using the default redundancy cut-off of 0.5 in KEGG pathways. Of these, we highlighted in Figure 6 the proteins associated to carbon metabolism (orange, *n* = 26), purine metabolism (light blue, *n* = 17), ribosome (pink, *n* = 19), aminoacyl-t-RNA biosynthesis (green, *n* = 16), biosynthesis of antibiotics (grey, *n* = 41) and amino acids (dark blue, *n* = 22).

## 4. Discussion

Endothelial cells (ECs) can be infected by several bacterial species, including *Rickettsia*, *Orientia*, *Ehrlichia* and *Anaplasma* (as reviewed in [31]). However, there are many gaps in the knowledge about how these pathogens interact with their host endothelial cells, and how this process can result in inflammation and endothelial dysfunction, leading to cardiovascular diseases [32].

**Figure 7:**
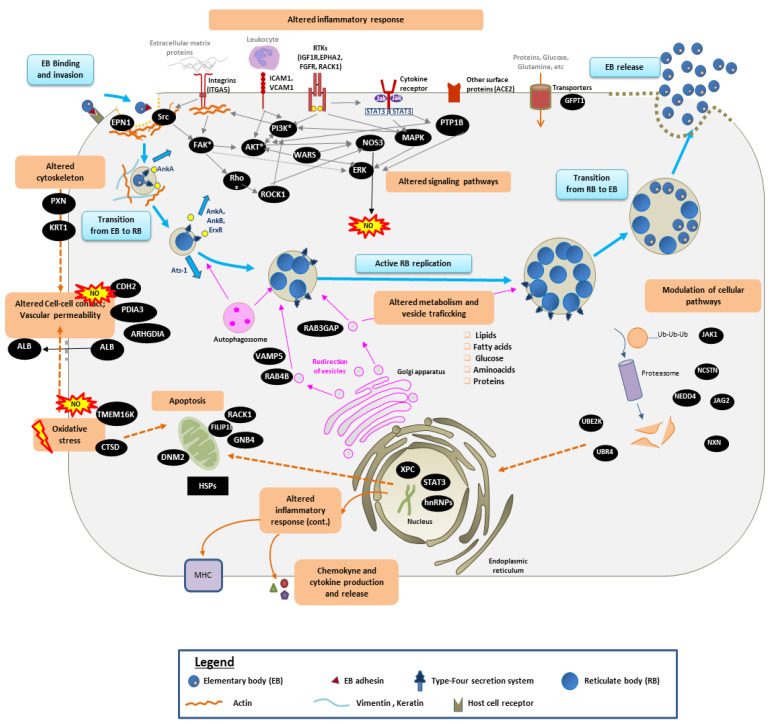
*E. ruminantium* infection model in BAE cells in vitro using proteomics. Different colors were used to highlight the bacterium life cycle and proteins (blue), the key changes in the host cells (orange), BAE surface proteins (drawn in red) and intracellular BAE proteins (black). This schematic drawing shows that *E. ruminantium* elementary bodies (EBs) (that have surface proteins such as adhesins) bind to an unidentified host cell receptor(s), triggering a hypothetical clathrin-mediated zippering process (involving EPN1, ITGA5 and Src proteins). This leads to an increase in the expression level of BAE cell surface proteins such as ICAM1 and VCAM1 (leading to endothelial cell activation and concomitant inflammatory response) and receptor tyrosine kinases (RTKs) (implicated in host cell signaling pathways such as JAK/STAT3, to prevent innate immune response). Downstream MAPK/Erk, PI3K/Akt/mTor and Notch/Wnt pathways are also modulated by the infection. After internalization into vacuoles, EBs differentiate into RBs, which divides via binary fission to form morulae. To establish a safe environment for active RBs replication, the bacterium recruits host molecule endosomal proteins (possibly Rab4B and VAMP5) to the vacuole, hampering fusion by host lysosomes. As the vacuole grows, cytoskeleton-associated proteins (such as Vimentin, keratin and actin) are remodeled to surround the inclusion membrane. The expression of tight junction-associated proteins (CDH2, PDIA3) is modulated to alter vascular permeability. *E. ruminantium* uses effector proteins such as AnkA, AnkB, ERxR and Ats-1 (which are translocated to host cytosol through the T4SS) to control the host cell mechanisms, such as apoptosis (avoiding host cell death) and ROS production (providing protection from oxidative damage). *E. ruminantium* dysregulates host cell vesicle trafficking (endosomes and autophagosomes) and cell surface transporters (GFPT1) to acquire nutrients (such as lipids, fatty acids, etc.) from the host cell. *E. ruminantium* also networks by targeting histone-modifying enzymes (such as nhRNPs, XPC, UBE2K and UBRA4) to promote intracellular survival. Following RBs transformation into EBs, these are released and spread to neighboring cells to start a new infection cycle. EB: Elementary body; RB: Reticulate body; AnkA: T4SS *E. ruminantium* effector homologous to AnkA; AnkB: T4SS *E. ruminantium* effector homologous to AnkB, ErxR: T4SS *E. ruminantium* effector homologous to ApxR; Ats-1: *E. ruminantium* effector homologous to Ats-1; T4SS: Type IV secretion system; EPN1: Epsin 1; Src: Tyrosine-protein kinase; ITGA5: Integrin alpha-5; ICAM1: Intercellular adhesion molecule 1; VCAM1: vascular cell adhesion molecule 1; IGF1R: Insulin-like growth factor 1 receptor; EPHA2: Ephrin type-A receptor 2 receptor A2; FGFR: Fibroblast growth factor receptor; RACK1: Receptor of activated protein C kinase 1; JAK: Janus kinase; STAT: signal transducers and activators of transcription; ACE2: Angiotensin converting enzyme; GFPT1: Glutamine-fructose-6-phosphate transaminase 1; FAK*: Focal adhesion kinase; Rho*: Rho family of GTPases; AKT*: protein kinase B; PI3K*: PI3 kinase; WARS: Tryptophan-tRNA ligase; ERK: extracellular signal-regulated kinase; NOS3: nitric oxide synthase; MAPK: mitogen-activated protein kinase; PTP1B: Protein tyrosine phosphatase non-receptor type 1 (also called PTPN1); PXN: Paxillin; KRT1: Keratin 1; NO: nitric oxide; CDH2: Cadherin 2; PDIA3: Protein disulfide-isomerase A3; ARHGDIA: Rho GDP-dissociation inhibitor; ALB: albumin; TMEM16K: Anoctamin, (ANO10); CTSD: Cathepsin; DNM2: Dynamin 2; HSPs: Heat-shock proteins; GNB4: G protein subunit beta 4; FILIP1L: Filamin A interacting protein 1 like; VAMP5: Vesicle-associated membrane protein 5; RAB4B: Ras-related protein Rab-4B; RAB3GAP: Rab3 GTPase-activating protein catalytic subunit; NCSTN: Nicastrin; JAG2: Delta-like protein; NEDD4: NEDD4 E3 ubiquitin protein ligase; NXN: Nucleoredoxin; UBE2K: Ubiquitin-conjugating enzyme E2 K; UBR4: Ubiquitin protein ligase E3 component n-recognin 4; XPC: XPC complex subunit; STAT3: Signal transducer and activator of transcription 3; hnRNPs: Heterogeneous nuclear ribonucleoproteins; MHC: major histocompatibility complex proteins; Ub: ubiquitination. * Indicates proteins present in BAE cells but not identified as differentially expressed in our study.

In this work, we analyzed the modulation of host and bacterial protein expression in Heartwater disease, using in vitro finite culture of endothelial cells (isolated from the aorta of bovines) and a virulent strain of *E. ruminantium* [5] and quantitative proteomics tools. We show that during the infection process, two dynamic players are interacting: (1) the bacterium, which aims to infect and replicate inside the EC (manipulating the host cell using bacterial effectors), and (2) the host ECs that readily interact with the pathogen (using cytokine and toll-like receptors) and fight back (using expression of adhesion molecules, chemokines and differential expression of MHC molecules) to induce an immune response. With this work, we propose the first hypothetical biochemical model of cellular infection by *E. ruminantium* (Figure 7), and we describe below which mechanisms may lead to *E. ruminantium*-induced pathobiology during the in vivo infection process.

### 4.1. Manipulation of the Host Immune Response and Signaling Pathways

Like monocytes and macrophages, ECs can detect and interact with foreign pathogens. For this, they express specific pattern recognition receptors (PRRs) which are triggered upon recognition of pathogen-associated molecular patterns (PAMPS) [32,33]. As for other *Ehrlichia* species [34], *E. ruminantium* does not express well-known PAMPs such as PG, pili, LPS and flagella or capsule. Therefore, the PAMP-triggered cytokine and chemokine production is likely to rely on the bacterium’s ability to modulate host cell signaling molecules upon infection.

The adhesion process for *E. ruminantium* has not yet been elucidated, but it is hypothesized that the binding of *E. ruminantium* to ECs cell receptors could be done through bacterial adhesins, such as the uncharacterized protein Q5FFC8 [9,35,36], Ankyrin-like protein Q5FH29 [37], AnkA [38] or the porin-like protein Q5FGU8 [36]. The proteins AnkA and Q5FGU8 were both detected in this work, but their roles in the infection process remain unknown.

Our results showed that BAE cell infection by *E. ruminantium* resulted in the over-expression of several ECs surface proteins, such as ICAM1, VCAM1, CSF1, ESM1 and MCAM. It is widely recognized that increased levels of ICAM1, VCAM1, ESM1 and MCAM activate ECs and induce the adhesion of circulating leukocytes to ECs, which is a key step in inflammatory responses of the innate immune system [39,40]. Overexpression of CSF1 could promote the release of pro-inflammatory chemokines and cytokines (such as IL-1/6/8, as described in [41]), thereby playing an important role in innate immunity and in inflammatory processes. This strongly suggests that BAE cells are indeed activated by *E. ruminantium* organisms, causing the first step of an inflammatory response. Similar results were obtained with other Rickettsiales species [42].

The infection process also led to the over-expression of other EC receptors involved in host–pathogen interaction (such as ITGA5 and ACE2), cell adhesion properties (for instance, SCARF1), protein transport (such as RHBDF2) and in downstream signaling pathways through EC receptors tyrosine kinases (RTKs). Overexpressed RTKs include IGF1R, EPHA2, FGFR1, 3 and 4, MET, PTPRA and PTPRU. The differential expression of RTKs such as RACK1, PTPN1, PTPN23, PTPRA and PTPRU, which are involved in JAK/STAT3 signaling, have a significant impact on downstream pathways, including MAPK/Erk (cell proliferation, survival, differentiation and migration) and PI3K/Akt/mTOr (apoptosis regulation, cell growth and cell cycle). This suggests that, as for other *Anaplasmatacae*, *E. ruminantium* can reprogram host cell-signaling pathways and manipulates ECs processes to create a beneficial environment for its replication. For instance, Wakeel and co-workers showed that *E. chaffeensis* effector TRP47 (the homologous protein of *E. ruminantium* Q5FF84) plays an important role in the inhibition of IFN-γ-induced tyrosine phosphorylation of Stat1, Jak1 and Jak2 by interacting with the protein tyrosine phosphatases PTPN2 [38]. Herein, we found that Jak1/STAT3 were only detected in the surface of infected cells, but we have no information on their tyrosine phosphorylation status. The protein tyrosine phosphatases PTPN1 (also called PTP1B) and PTPN23 were detected in infected cells only. The elevated PTP1B expression can promote cell proliferation via activation of protein kinase B (AKT), extracellular signal-regulated kinase (ERK) 1/2 and focal adhesion kinase signaling pathways, and these are associated with angiogenesis, cytoskeletal reorganization and shear stress-responsive gene expression. As such, PTP1B has been considered as a potential therapeutic target to promote neovascularization in ischemic cardiovascular diseases [43].

We also detected other proteins such as DIPK2A (detected only in infected cells) and WARS (proteoforms over- and under-expressed), which are known to regulate ERK, AKT and NOS3 activation pathways. The over-expression of ICAM-1 (mentioned above) can induce AKT phosphorylation in a PI3K-dependent manner. The consequent activation of AKT is known to lead to NO production [44], a free radical participating in the inflammatory processes. The FGFR/FGF signaling is known to activate the PI3K/Akt/ mTOR pathway [45]. Herein, we detected members of the FGFR family such as FGFR1, FGFR3 and FGFR4 (all overexpressed in infected cells), which are frequently amplified or overexpressed in diseases. The combination of FGFR inhibitors with inhibitors of the PI3K/AKT/mTOR pathway has been considered as an effective strategy for clinical development [45].

The Notch and Wnt cell signaling pathways are also affected by *E. ruminantium* infection. These pathways represent two major networks of communication used by eukaryotes to control cell fate (proliferation, differentiation and apoptosis). It has been recently shown that *E. chaffeensis* uses ehrlichial TRPs to exploit both Notch and Wnt host cell signaling pathways to downregulate innate immune host defenses and promote ehrlichial internalization and infection [46,47,48]. Herein, we detected Wnt signaling proteins, such as NXN (detected only in uninfected cells), WDR61 and CCNY (detected only in infected cells) and GNB2L1 (under-expressed). We also observed the modulation of key proteins involved in the Notch pathway, such as: JAG2 (putative Notch ligand, only in infected cells)*,* AAK1 and NEDD4 (both being under-expressed). NEDD4 E3 ligase is an important regulator of Notch signaling, and it ubiquitinates Notch and regulates Notch expression at the cell surface, causing its removal from the cell surface and targeting it for lysosomal degradation [49]. Nicastrin (NCSTN), that plays a role in Notch and Wnt signaling cascades, was found to be overexpressed in infected cells. How the Wnt and Notch pathways are exploited to promote *E. ruminantium* survival remains to be determined. Herein, we detected in the *ER* elementary body phase the T4SS bacterial effectors homologous to AnkA, AnkB and ApxR (now called ErxR [50]), which are known to interfere with several host cell signaling pathways in *Anaplasma* infection [51,52]. It is possible that these effectors were not detected in the replicative form of the bacterium (RBs) due to high amounts of host contaminants (indeed, no purification procedures were used for RBs).

Another way ECs respond to the infection is by altering the expression of enzymes associated to important antioxidant defense mechanisms. Reactive nitrogen (such as nitric oxide, NO) and oxygen species (such as superoxide and hydrogen peroxide) have been implicated in the relationship between mammalian hosts and microbial pathogens. For instance, infection of human ECs by *R. rickettsii* results in a significant reduction in the levels of key enzymes involved in protection against oxidative injury [53]. *E. chaffeensis* takes advantage of combined mechanisms to prevent ROS damage to itself and to host cells [54]. Herein, we observed, in infected cells, (i) the over-expression of enzymes such as HMOX2, NOS3, ANO10 (also known TMEM16K), DLDH and PTGS1, and (ii) the under-expression or absence of enzymes such as GLRX3, NXN, TXN, PRDX 2, 4 and 6 (all members of the TRX system) and G6PD. The over-expression of enzymes such as HMOX2, PTGS1 and TMEM16K can have a substantial influence on endothelial inflammatory responses and vascular permeability. This result provides a mechanistic explanation into how *E. ruminantium* infection of the endothelium leads to vasodilation and fluid leakage into the interstitial spaces. Due to the great importance of these proteins [55], modifications of TMEM16 proteins have been suggested as a novel therapeutic strategy for endothelial dysfunction-associated diseases [56].

Previous reports demonstrated that infection of EC with *E. ruminantium* resulted in induction of NO production, and that excessive NO production can have an important role in resistance to *E. ruminantium* infection [41,57]. Herein, we observed increasing levels in NOS3 protein, throughout the bacterial life cycle. Besides its antimicrobial activity, NO has several key functions, affecting the expression and activity of Rho GTPases, regulating cell motility and the actin cytoskeleton.

The TRX system is another antioxidant defense mechanism affected by *E. ruminantium* infection. The TRX system, in conjunction with glutathione and GLRX3, maintains the reducing environment of the cell and detoxifies ROS [58]. We found in this work a major downregulation of the TRX system family proteins. The absence of TXN and PDRX-2, 4 and 6 suggests that *E. ruminantium* can control the release of H_2_O_2_ from the host cells. The reasons for this phenomenon are unclear, and we hypothesized that the lysosomal aspartic proteinase Cathepsin (also known as CTSD) may alter Trx protein levels (which is an essential anti-apoptotic and reactive oxygen species scavenging protein in endothelial cells) and thereby promote formation of ROS and apoptosis. Whether cathepsin (over-expressed here) plays a role in host response to *E. ruminantium* infection may be an important direction for future research. The decrease in G6PD in infected cells can also have an impact on the conversion of toxic peroxides to innocuous by-products [59], and hamper the repair of redox balance in infected endothelial cells.

Interestingly, despite this highly oxidative environment caused by its own infection process, *E. ruminantium* can successfully replicate in ECs. It has been demonstrated that *E. chaffeensis* can replicate in macrophages (which are producers of ROS) by actively inhibiting superoxide generation [60], and the mechanism underlying this phenomena would involve iron and the two-component systems (TCS) CckA-CtrA [61]. The TCSs (which are important to bacteria to sense their external environment) have been previously detected in *E. chaffeensis* and *A. phagocytophilum,* and it has been suggested that these proteins would be active mostly during the bacterial intracellular development [61]. Proteins of the TCSs were previously detected in *E. ruminantium* elementary bodies [5]. Here, we have detected for the first time 3 response regulators, CtrA, PleD and NtrX, in actively replicating *E. ruminantium* reticulate bodies. We also noticed that *E. ruminantium* reticulate bodies produce several chaperones (such as DnaK, DnaJ, HslU, GroEL, GroES and HtpG proteins) and other key proteins involved in cell homeostasis/oxidative stress response (such as TrxB, ElbB, Sco2, TsaA PepA, ClpX, ClpB and Lpd). It should be mentioned that, in *A. phagocytophilum* infection, Lpd can act as an immunopathological molecule, affecting cytokine and chemokine production [62]. As ehrlichial Lpd was detected as early as 48 hpi in infected cells, this could explain, at least in part, the acute clinical signs observed in vivo during the infection with *E. ruminantium*. Globally, these results support the idea that *E. ruminantium* reticulate bodies can cope with ECs redox state, without affecting their replicative process.

### 4.2. Remodeling of the Cytoskeleton

The entry process of *E. ruminantium* has not yet been studied. Our results suggest that *E. ruminantium* internalization could be performed via a clathrin-mediated zippering process, using cellular integrins and cadherins. Indeed, in *E. ruminantium*-infected BAE cells, we detected the presence of the ‘classic’ cadherins such as neuronal (N) adherin (also known as CDH2) and 3 protocadherins (PCDH17, PCDHGA2 and PCDHGC3), all involved in cell adhesion. The role for clathrin-dependent endocytosis was already described for other Rickettsiales, such as *R. conorii* and *Neorickettsia risticii* [63]. The overexpression of EPN1, that binds to membranes enriched in phosphatidylinositol 4,5-bisphosphate (PtdIns(4,5)P2) and modifies membrane curvature and facilitates the formation of clathrin-coated invaginations, could also be involved in this process.

Bacterial adhesins can form a bridge with cellular integrins (such as ITGA5), inducing integrin clustering and signaling in cells [64]. This would induce a host cell invasion via Src tyrosine kinase activation; here, Src was found to be detected only in infected cells. Other molecules that regulate the establishment of integrin-containing contacts are also affected: for instance, the downregulation of the protein RAB4B may result in lower integrin association with the cytoskeleton via phosphatydylinositol 4-phosphate 5-kinase/PIP2/vinculin and talin conformational changes. Focal adhesion-specific protease calpain (which controls both turnover and activation of focal adhesion components) was found to be downregulated. We also noticed the overexpression of PTPRA and Ociad1 in infected cells, both involved in integrin-mediated focal adhesion formation. Similar results were observed with spotted fever group rickettsiae [65]. Cell–cell contact is also affected by *E. ruminantium* infection at several levels of contact: (i) adherents’ junctions (AJs) (CDH2 and CTNNA3 were detected only in infected cells), (ii) tight junctions (JAM3 was detected only in non-infected cells, while SYMPK was found only in infected cells) and (iii) desmosome (PNN was found to overexpressed in infected cells). It is worth mentioning that the modification of cytoskeletal proteins and/or tight junction proteins can lead to vascular permeability and to a redirection of albumin transport to peripheral tissues [66], and this could explain the downregulation of albumin (Alb) in infected cells.

*E. ruminantium* internalization might be facilitated by additional mechanisms. We detected a modulation of PDIA3 proteoforms (either over- or under-expressed depending on proteoforms), and this protein is known to be implicated in the invasion process for many *Chlamydia spp.* [67] and therefore could have an important role in *E. ruminantium* adhesion and internalization. We also detected a modulation in ARHGDIA and ARHGDIB proteins. Since ARHGDIA is the negative regulator of Ras-related Rho family of GTPases (key regulators of many cellular processes involved in cell transformation), we hypothesize that the decreased expression of ARHGDIA may lead to the activation of Rho family GTPases during vascular remodeling [68,69]. The overexpression of the FGD5 protein and consequent activation of CDC42 (a member of the Ras-like family of Rho and Rac proteins) could also be implicated in this process. We also found that the Rho kinase ROCK1 (which functions as a suppressor of neutrophil and macrophage migration in vivo [70]) was detected only in *E. ruminantium*-infected cells, suggesting that this protein is essential for *E. ruminantium* infection. In fact, it has been previously shown that mammalian cells with reduced levels of ROCK1 were not as permissive to *A. phagocytophilum* [71]. The use of inhibitors of ROCK1 are being considered in the treatment of a number of human diseases (including human anaplasmosis), as non-antibiotic-based strategies to combat infection (as described in [71]).

As observed in other bacterial infection [39,72,73], cytoskeletal modifications and the formations of gaps between ECs might also contribute to *E. ruminantium* internalization. Herein, we observed the differential expression of several EC cytoskeleton-related proteins, such as ACTG1 (over-expressed), myosin (MYO1D, under-expressed, MYO5A and MYO9B, both over-expressed), fibronectins (FNDC3A and FNDC3B, over-expressed), KRT1 (with proteoforms being over- or under-expressed), TBB5 (under-expressed) and VIM (with proteoforms being over- or under-expressed). We also detected a modulation in the expression of several EC proteins required for actin filament dynamics and organization. For instance, the proteins FMNL3, EPB41L1, SSH2, CORO1B and ARPC2 are detected only in *E. ruminantium*-infected cells, while in FSCN1 and GC, proteins are under-expressed or inhibited by infection. The modulation of such proteins can have a significant impact in cell–cell adhesion and cell–extracellular matrix adhesion. For instance, proteins FERMT2, PXN and the adapter protein CrkII (which are key proteins involved in the assembly of focal adhesion protein complex and actin cytoskeleton remodeling) were found to be inhibited or under-expressed in *E. ruminantium*-infected cells.

Collectively, these results strongly suggest that the interaction between *E. ruminantium* and ECs could ease *E. ruminantium* internalization and allow the bacterial inclusion to grow during the replication of reticulate bodies but could also result in increased vascular permeability. This last feature would be responsible for the generalized circulatory failure, seen as lung congestion, hydrothorax and hydropericardium, that is observed in Heartwater infection. This is supported by the expression of other various vasoactive agents secreted by EC, such as the vasodilatory NO (over-expression of the enzyme NOS3), as well as the vasoconstrictor endothelin I (we detected the enzyme Endothelin converting enzyme 1 (ECE1) only in infected cells) and angiotensin II (Angiotensin converting enzyme (ACE) only in infected cells).

### 4.3. Manipulation of Metabolism and Vesicle Trafficking

To replicate inside the host cells, *E. ruminantium* needs metabolites such as amino acids, carbohydrate and lipids. *E. ruminantium* genomic information describes an aerobic organism that probably does not ferment carbohydrates, such as glucose [7,74]. The main carbon sources are likely to be proline and glutamate [75,76]. In *E. ruminantium* genome, all enzymes of the tricarboxylic acid (TCA) pathway were found, accompanied by a putative glutamate dehydrogenase, which would facilitate the transfer of glutamate into the TCA cycle. There is a partial gluconeogenesis pathway, terminating at fructose-6-phosphate, as well as a complete non-oxidative pentose-phosphate pathway. *E. ruminantium* has genes for the biosynthesis of only five amino acids (arginine, lysine, proline, glutamate and glutamine). Proteomics experiments on *E. ruminantium* elementary bodies confirm the expression of many of the predicted enzymes for amino acid metabolism, the TCA cycle, a partial gluconeogenesis pathway and a complete non-oxidative pentose-phosphate pathway. In the virulent strain, we detected a higher number of proteins involved in energy conversion and production. This energy could be used to fuel specific processes, namely those involved in virulence [5,9]. Herein, we detected for the first time RB-associated proteins related to amino acids and proteins’ biosynthesis, glycolysis, fatty acid and lipid biosynthesis, co-factor biosynthesis and proteins related to DNA replication (which is in agreement with previous transcriptomics results [6,8]). Still, *E. ruminantium* is unable to synthesize the cell wall components, as other *Anaplasmataceae*. Hence, these are probably obtained from the host cell.

The transport of lipids and proteins is a complex process in mammalian cells, involving vesicular and non-vesicular pathways [67,77]. *E. chaffeensis* [46] and *A. phagocytophilum* [78] have developed fine-tuned strategies to deviate the fate of the transporting vesicles for their own use, while avoiding lysosomal degradation of their own replicating vacuoles. For instance, *A. phagocytophilum* uses its bacterial effector Ats-1 to recruit autophagosomes to its replication vacuole to obtain nutrients [79]. Herein, we also detected the *E. ruminantium* effector Ats-1, and it is possible that *E. ruminantium* uses autophagy for this purpose. *E. chaffeensis* bacterial inclusion expresses early endosome-like characteristics (such as transferrin, transferrin receptor, the small GTPase RAB5) and early autophagosome markers (such as VPS34, the catalytic subunit of class III phosphatidylinositol 3-kinase (PI3KC3), and ATG5, the autophagy double-membrane initiation protein) [80]. The composition of *E. ruminantium* morula is currently unknown. However, we found evidence of autophagy modulatory function by the RAB3GAP1 (together with RAB3GAP2). Besides being used for nutrient acquisition, autophagy is also used as an innate immune response pathway. A recent study showed that, although autophagy is usually induced during bacterial infection, *Ehrlichia* appears to be able to inhibit autophagy during its infection process. Although no detailed studies have been performed to understand how *Ehrlichia* inhibits autophagy, the role for the functional two-component (TC) system in the inhibition of phagosome lysosome fusion during ehrlichial infection has been reported [81]. We also noticed the presence of porins and transport-associated proteins in the RB, and these would probably be very useful to scavenge for metabolites from the host cells, while controlling them through the use of effector secreted by the T4SS (herein, we detected virB). We also detected a Patatin-like protein (Q5FH82, homologous to *E. chaffeensis* EchaDRAFT_0464). This intracellular cytotoxin is known to disturb membrane trafficking and regulate intracellular bacterial growth [82], and it could therefore promote *E. ruminantium* intracellular replication in BAE cells.

*E. chaffeensis* [46] and *A. phagocytophilum* [78] can manipulate their host cells to acquire cholesterol and other lipids. Herein, we noticed that *E. ruminantium* interferes with both vesicular and non-vesicular pathways to acquire lipids. We observed an upregulation of 3-hydroxy-3-methylglutaryl coenzyme A synthase (HMGCS1) and FDFT1 protein, both involved in cholesterol biosynthesis, and we also observed the inhibition of Oxidized low-density lipoprotein receptor 1 (OLR1). This suggests a de novo lipid biosynthesis in host cells, and cholesterol is then incorporated into the *E. ruminantium* inclusion bodies. We also observed the differential expression of BAE proteins associated to other lipids and fatty acid metabolism, namely the under-expression of FASN, FABP4 and FAPB5 and the production of the proteins FAR2, ACOX1 (Fatty-acid beta-oxidation) and ECHS1. We also detected the over-expression of dynamin-family proteins, DNM2 and EHD4, which are GTPase mechanoenzymes involved in cellular functions of membrane tabulation and scission, vacuole fission and fusion, peroxisome maintenance, endocytosis and intracellular trafficking [83]. Non-vesicular mechanisms involve lipid transporters, including the ceramide endoplasmic reticulum transport protein (COL4A3BP, present only in infected cells) and members of the high-density lipoprotein (HDL) biogenesis machinery, which deliver host phosphatidylcholine. The acquisition of glycerophospholipids requires the activation of phospholipase A2 (PRDX6, over-expressed) and mitogen-activated protein kinase (MAPK). These pathways participate in the sequestration of host lipid metabolites, and could be used by *E. ruminantium* for both regulation of immune responses and energy production [84].

### 4.4. Inhibition of Cell Apoptosis

Intracellular bacteria usually require several days of intracellular replication. As such, it is plausible that, as for other obligate intracellular bacteria [34,67,69] *E. ruminantium* might prolong host cell viability, by actively inhibiting apoptotic signaling pathways and/or inducing pro-survival factors. Herein, we found several indications that support this idea. For instance, the EC receptor EPHA2 was found to be overexpressed throughout the *E. ruminantium* replication cycle. In *C. trachomatis* serovar L2 infection, this receptor induces PI3 kinase (PI3K) activation, promoting normal chlamydial replication [85]. The proteins RACK1 (also called GNB2L) and GNB4 were found to be differentially expressed. RACK1 (which promotes apoptosis) is under-expressed and GNB4 (which contributes to the tight balance between proapoptotic (BAX) and antiapoptotic (BCL2) proteins) is detected only in infected cells. Interestingly, FILIP1L was found to be over-expressed in infected cells at 72 hpi, and it is known that when over-expressed in EC, it inhibits cell proliferation and migration, and leads to an increase in cell apoptosis.

One of the hallmarks of apoptosis is the degradation and compaction of chromatin [86]. Our results suggest that *E. ruminantium* can disturb chromatin condensation, as it modulated the expression of several chromatin-associated proteins, such as the histone H4, MCM7, HNRNPF and HNRNPH1 (all under-expressed in infected cells), BABAM2 and POLR1C (detected only in non-infected cells) and XPC complex subunit DNA damage recognition and repair factor (only in infected cells). Histone levels can be controlled by the ubiquitin-proteasome system [87]. Ubiquitin proteins UBE2K and UBR4 were found to be detected in non-infected cells and under-expressed in infected cells, respectively.

Cell survival and apoptosis are also linked to morphological changes in mitochondria. Overexpression of YME1L1 is known to promote apoptosis [88], and this mitochondrial protein was found to be detected only in *E. ruminantium*-infected cells. Herein, we found that DNM2, that is critically involved in mitochondrial fission, was detected in infected cells [89]. Cells can also produce high levels of heat-shock proteins (HSPs) to protect themselves against apoptosis [90]. Furthermore, HSPs were found to be highly expressed in cardiovascular tissues, and to induce inflammatory responses. Herein, we detected the differential expression of 8 HSPs: HS71B (under-expressed), HSP90B1 (also called endoplasmin, over-expressed), HSPA5 (over- and under-expressed depending on the proteoform), HSPA6 (under-expressed), HSPA8 (over-expressed), HSPA9 (over- and under-expressed depending on the proteoform), HSPB1 (over- and under-expressed depending on the proteoform) and HSPD1/HSP60 (over-expressed). The increase in HSP60 levels was also observed for other bacteria such as *Chlamydia*, with immunopathogenic consequences [91].

Despite this harsh environment, *Anaplasmatacae* can resist to apoptosis. For instance, *E. chaffensis* uses its T4SS effector ECH0825 to inhibit human Bax-induced apoptosis [92]. Its counterpart *Anaplasma* translocated factor 1 (Ats-1) is secreted by the T4SS and was recently shown to be responsible in part for the antiapoptotic effects of *A. phagocytophilum* [93]. Ats-1 also inhibits the docking of human Bax to mitochondria and the subsequent apoptosis of yeast, which lacks Bcl-2 members, after the induction of the human Bax protein (166). Herein, we detected the Ats-1 homologous in *E. ruminantium* at 96 (reticulate body) and 120 hpi (elementary body), which strongly suggest implication of this anti-apoptotic factor in maintaining host cell integrity during the differentiation of RBs into infective EBs.

Other biochemical features important in host cell manipulation by *E. ruminantium*. Various examples exist where pathogens target, manipulate and take advantage of host PTMs to facilitate their survival strategy [94]. Our group already showed that *E. ruminantium* is able to modify this machinery [5,6,8], and we demonstrated the importance of glycosylation and phosphorylation of bacterial proteins in the virulence of *E.ruminantium* [9]. Here, we detected a downregulation of GFPT1, which controls the flux of glucose into the hexosamine pathway, and, therefore, the formation of hexosamine products and the availability of precursors for N- and O-linked glycosylation of proteins. A decrease in GFPT1 in ECs suggest hypoglycosylation and, consequently, a defective function of several ECs proteins, together with a highest availability of glucose for *E. ruminantium* metabolism and replication.

Besides the *E. ruminantium* proteins discussed above, we also detected others present in the metabolically active reticulate body. The role of the T4SS in the pathogenicity life cycle of bacterial pathogens is well-established, including in other *Rickettsiales,* such as *A. marginale*, *E. chaffeensis* and *E. canis* (as reviewed in [51,95,96]. Here, we detected 4 out the 10 “building blocks” of the *E. ruminantium* T4SS (VirB10, B11, B4 and VirD4, Supplementary Table S6). We also detected several membrane-associated proteins, such as the MAP1-protein family, porins, transporters and uncharacterized proteins, whose effective functions remain to be elucidated.

## 5. Conclusions

Our study provides an integrated host and bacterial proteomics analysis of the infection of primary bovine endothelial cells with the etiologic agent of Heartwater, *E. ruminantium*. Our experimental design was intended to collect infected endothelial cells once they were homogeneously infected, to mimic a vascular infection due to *E. ruminantium* dissemination. We used proteomics tools and bioinformatics analyses to identify the pivotal proteins and pathways that differ between infected and non-infected cells during the *E. ruminantium* infectious cycle. We also presented for the first time major proteins present in RB, during the active replication of *E. ruminantium*. In an attempt to ease the understanding of the role of the DEPs, we tried to separate them by pathways, but it should be mentioned that many of these proteins can have proteoforms (PTMs can affect over- and under-expression), can participate (directly or indirectly) in several pathways and they are globally interlinked (as we showed using Cytoscape and in Figure 7).

Globally, our results reveal that *E. ruminantium* induces the modulation of a wide range of host cellular pathways, such as metabolite acquisition, molecular trafficking, cell membrane, cytoskeletal rearrangement and host immune response modulation. Our results indicate that BAE cells recognize *E. ruminantium* antigens through cell surface receptors (such as VCAM-1, ICAM-1 and integrins) and cytosolic innate immune sensors. Activation of these receptors triggers the release of pro-inflammatory cytokines and chemokines, which recruit inflammatory cells. However, this inflammatory response, which is required for bacterial clearance, is also responsible for several aspects of the clinical pathobiology of Heartwater, including endothelial leakage, microvascular hemorrhage and multiorgan failure.

With the improvement of effective genetic tools for the manipulation of intracellular bacteria [97], but despite the lack of effective genetic tools to functionally analyze *E. ruminantium,* many fundamental research questions concerning *E. ruminantium*–host interaction can now be approached by using cell biological techniques, the targeted knockdown of genes in mammalian cell culture, for instance using RNAi. Such technologies will help in completing the puzzle that we started to build on the molecular interactions that govern the infectious cycle of *E. ruminantium* in its host cell.

## Figures and Tables

**Table 1:** Summary of differentially expressed BAE and *E. ruminantium* proteins during the bacterium life cycle.

	Samples for Analysis		BAE Proteins				*E. ruminantium* Proteins
			(Includes Isoforms)				
Status	Infected BAE Cells	Non-Infected BAE Cells	Over-Expressed (Ratio Infected/Non-Infected)	Under-Expressed (Ratio Infected/Non-Infected)	Only in Infected Cells	Only in Non-Infected Cells	Only in Infected Cells
**2D-DIGE-MS/MS**	24–48–72 hpi		84	100	-	-	-
**1DE-nLC-MS/MS**	24–48–72–96–120 hpi		14	54	36	27	265
**Biotin-UPLC-MS/MS**	72 hpi		-	7	104	7	-
**Total**	24–48–72–96–120 hpi		98	161	140	34	265

## Supplementary Materials

The following are available online at https://www.mdpi.com/article/10.3390/microorganisms9061144/s1. Figure S1. Overall workflow for integrated profiling of the proteomes of bovine aortic endothelial cells (BAE) and *Ehrlichia ruminantium* strain Gardel virulent (ER) along the bacterium life cycle. “hpi”: hours post-infection. Figure S2. 2D-DIGE experiments. Representative gels with selected spots of interest (A) and results from Image analysis using Decyder software, where Spot maps evidence 2 distinct groups in the experiments: *Ehrlichia ruminantium* infected and non-infected BAE cells (B). Table S1. Proteins differentially expressed between *Ehrlichia ruminantium* infected and non-infected BAE cells at 24, 48 and 72 hpi, according to 2D-DIGE-MS/MS (data compiled using Protein-Pilot software). Table S2. Proteins differentially expressed between *Ehrlichia ruminantium*-infected and non-infected BAE cells along the bacterium life cycle (from 24 to 120 hpi) according to 1DE-nLC-MS/MS (data compiled using MaxQuant and Perseus software). Table S3. Proteins differentially expressed between *Ehrlichia ruminantium*-infected and non-infected BAE cells at 72 hpi according to biotin-nanoUPLC-MS/MS (data compiled using ProteinLynx Global SERVER). Table S4. List of proteins differentially expressed between *Ehrlichia ruminantium*-infected and non-infected BAE cells (according to the results obtained by 2D-DIGE-MS/MS, to 1DE-nLC-MS/MS and biotin-nanoUPLC-MS/MS) utilized to perform an integrated data analysis with Cytoscape. Table S5. List of GO terms and Reactome for the proteins differentially expressed between *Ehrlichia ruminantium*-infected and non-infected BAE cells (according to the results obtained by 2D-DIGE-MS/MS, to 1DE-nLC-MS/MS and biotin-nanoUPLC-MS/MS and Cytoscape integrated analysis). Table S6. *Ehrlichia ruminantium* proteins differentially expressed along the bacterium life cycle (from 24 to 120 hpi) according to 1DE-nLC-MS/MS (data compiled using MaxQuant and Perseus software).
