# Supplementary figures and images for: Revisiting Ehrlichia ruminantium Replication Cycle Using Proteomics: The Host and the Bacterium Perspectives
Source: Microorganisms. 2021 May 26;9(6):1144. doi: 10.3390/microorganisms9061144 (PMC8229282; doi:10.3390/microorganisms9061144)

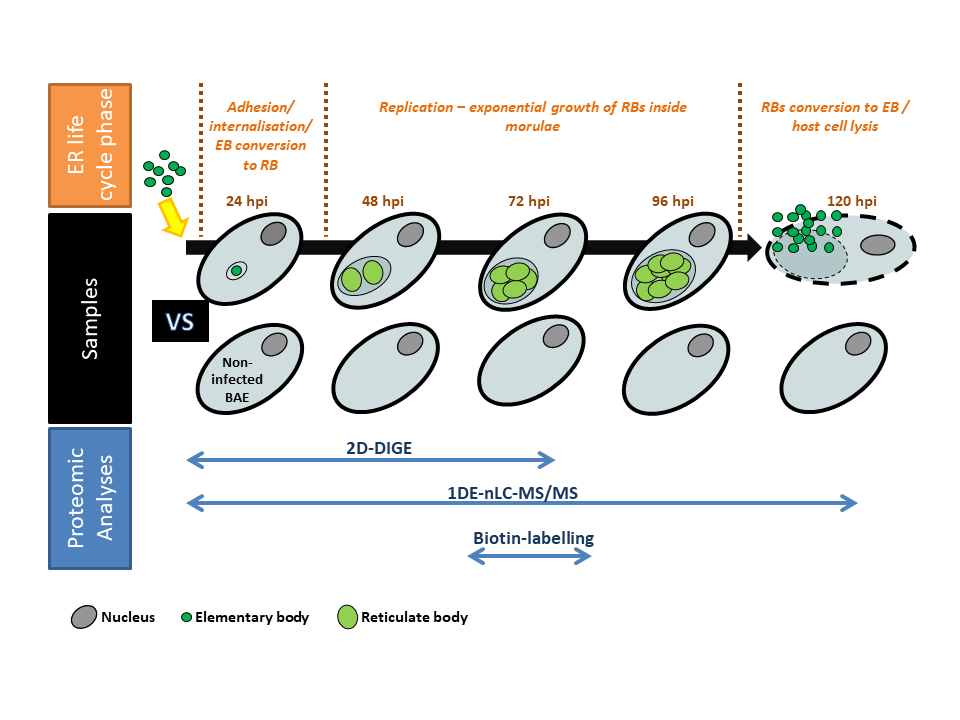

Supplement: Supplementary file 1 [file microorganisms-09-01144-s001.zip › supplemental data 22may2021/Figure S1.png]

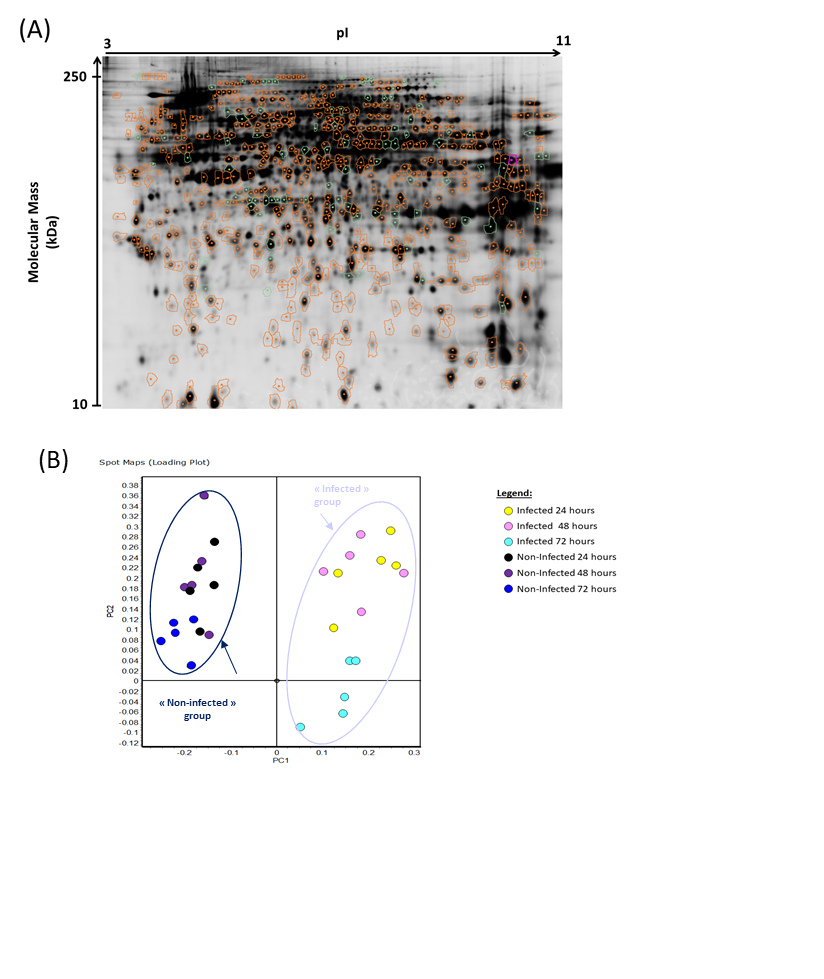

Supplement: Supplementary file 1 [file microorganisms-09-01144-s001.zip › supplemental data 22may2021/Figure S2_final_27april2021.tif]
